# Supplementary material for: The Omicron variant BA.1.1 presents a lower pathogenicity than B.1 D614G and Delta variants in a feline model of SARS-CoV-2 infection
Source: bioRxiv. 2022 Jun 16:2022.06.15.496220. Preprint. [Version 2] doi: 10.1101/2022.06.15.496220 (PMC9216722; doi:10.1101/2022.06.15.496220)
Supplement: 1 [file NIHPP2022.06.15.496220V2-supplement-1.pdf]

(which was not certified by peer review) is the author/funder, who has granted bioRxiv a license to display the preprint in perpetuity. It is m
available under aCC-BY 4.0 International license.

Supp. Fig. 1

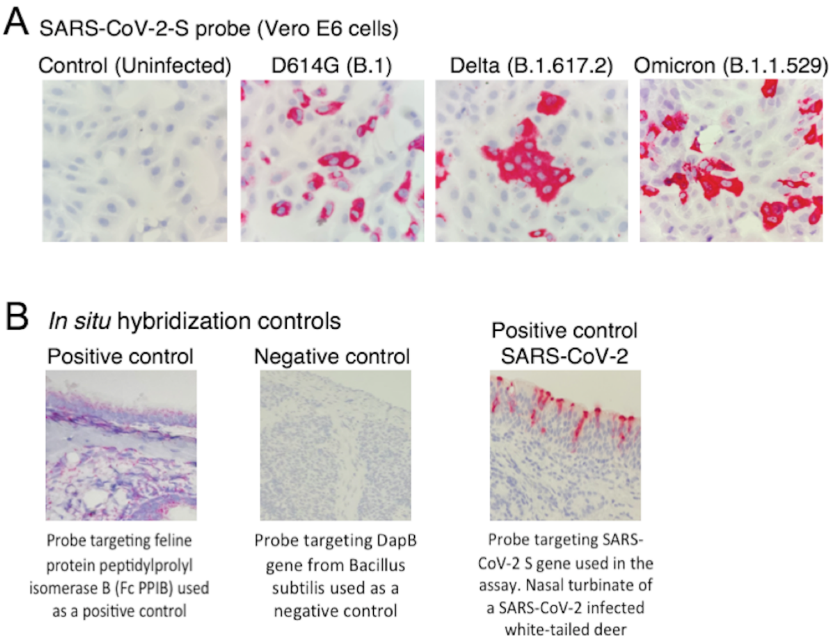

Suppl. Fig.1 *In situ* hybridization (ISH) controls. Vero E6 cells were inoculated with SARS-CoV-2 D614G (B.1 lineage) (isolate NYI67-20), Delta (B.1.617.2 lineage) (isolate NYI31-21), and the Omicron BA.1.1 (B.1.1.529) (isolate NYI45-21) at a multiplicity of infection (MOI) of 0.1. Cells were fixed 24 h post-infection (h pi) and subjected to subjected to ISH using the RNAscope® 2.5 HD Reagents–RED kit (Advanced Cell Diagnostics) following the manufacturer's instructions using a probe targeting SARS-CoV-2 RNA spike (V-nCoV2019-S probe ref # 848561). Slides were counterstained with hematoxylin; 40x magnification (**A**). ISH using the RNAscope® 2.5 HD Reagents–RED kit (Advanced Cell Diagnostics) following the manufacturer's instructions. Left panel, nasal turbinate of a cat using a probe targeting feline host protein peptidylprolyl isomerase B (PPIB) was used as a positive control (Advanced Cell Diagnostics cat # 455011). Middle panel, nasal turbinate using a probe targeting DapB gene from Bacillus subtilis strain SMY was used as a negative control (Advanced Cell Diagnostics cat # 310043). Right panel, nasal turbinate of white-tailed deer infected with SARS-CoV-2 B.1 D614G using a probe targeting SARS-CoV-2 RNA spike (V-nCoV2019-S probe ref # 848561) was used as positive control. Slides were counterstained with hematoxylin; 40x magnification (**B**).

(which was not certified by peer review) is the author/funder, who has granted bioRxiv a license to display the preprint in perpetuity. It is m
available under aCC-BY 4.0 International license.

Supp. Fig. 2

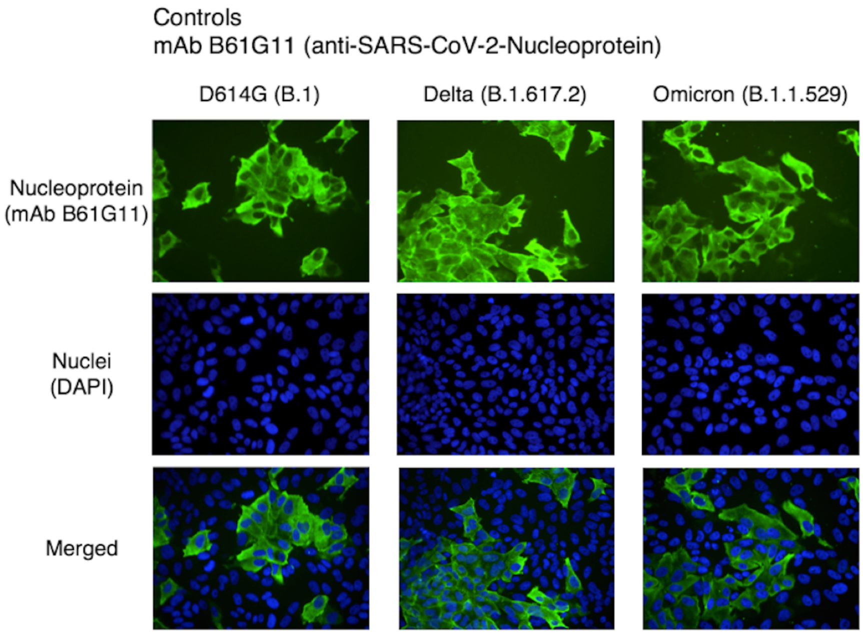

Suppl. Fig.2 *In situ* immunofluorescence (IFA) controls. Vero E6 cell were inoculated with SARS-CoV-2 D614G (B.1 lineage) (isolate NYI67-20), Delta (B.1.617.2 lineage) (isolate NYI31-21), and the Omicron BA.1.1 (B.1.1.529) (isolate NYI45-21) at a MOI of 0.1. Cells were fixed 24 h pi and subjected to an immunofluorescence assay using a monoclonal antibody (B61G11) anti-SARS-CoV-2-nucleoprotein (N) (Green). Nuclear counterstain was performed with DAPI (Blue); 40x magnification.
